# Fibromatosis Colli Presenting as a Left-Sided Cervical Mass: A Case Report

**DOI:** 10.7759/cureus.115519

**Published:** 2026-08-31

**Authors:** Daniel Jennings, Courtney Shockley

**Affiliations:** 1 Pediatrics, Pacific Northwest University of Health Sciences, Yakima, USA; 2 Pediatrics, Nourish Wellness, Yakima, USA

**Keywords:** computed tomography, congenital muscular torticollis, fibromatosis colli, neonatal neck mass, physical therapy, sternocleidomastoid pseudotumor, ultrasonography

## Abstract

Fibromatosis colli is a rare, benign condition of infancy characterized by fibrous enlargement of the sternocleidomastoid (SCM) muscle. It typically presents during the first several weeks of life as a painless cervical mass and may be associated with congenital muscular torticollis (CMT). Ultrasonography is the preferred initial imaging modality; however, variable sonographic appearances may complicate recognition.

A two-week-old female patient, with an uncomplicated neonatal course, presented for a routine well-child examination and was found to have a new, painless, left-sided cervical mass. Initial ultrasonography demonstrated a 2.1 × 1.5 × 2.5 cm circumscribed structure with internal Doppler vascularity and no visible fatty hilum, raising concern for a solid lesion or abnormal lymph node. Contrast-enhanced computed tomography demonstrated fusiform enlargement of the left SCM without a discrete cervical mass or lymphadenopathy, consistent with fibromatosis colli. Subsequent review of the initial ultrasound at a tertiary pediatric center was also considered consistent with the diagnosis. At approximately seven weeks of age, rightward head rotation and leftward head tilt consistent with CMT were documented, and the patient was managed conservatively with physical and occupational therapy. At approximately 4.5 months following diagnosis, parental report indicated equal bilateral head rotation and achievement of multiple age-appropriate developmental goals.

This case highlights several clinically relevant features of fibromatosis colli. The condition may present after an initially normal newborn examination, and its variable sonographic appearance may create diagnostic uncertainty. Recognition of the relationship between the sonographic abnormality and the SCM may improve diagnostic confidence and potentially avoid additional imaging with ionizing radiation. Continued clinical surveillance is important because CMT may become apparent after the initial presentation and can be managed conservatively.

## Introduction

Fibromatosis colli, also known as sternocleidomastoid pseudotumor of infancy, is a rare, benign condition characterized by fibrous enlargement of the sternocleidomastoid (SCM) muscle. It has been reported in approximately 0.4% of newborns and classically presents as a firm, painless cervical mass during the first several weeks of life [1,2]. Although its pathogenesis remains incompletely understood, proposed mechanisms include intrauterine mechanical factors, perinatal injury, vascular compromise, and subsequent fibrosis of the SCM [1,2]. However, many affected infants have no history of complicated delivery, suggesting that birth trauma alone does not adequately explain its development [2].

Fibromatosis colli may present as an isolated cervical mass or in association with congenital muscular torticollis (CMT). Although closely associated, fibromatosis colli and CMT are not synonymous; fibromatosis colli refers to the SCM lesion, whereas CMT describes the associated clinical postural abnormality. Because a cervical mass in a neonate raises consideration of congenital, inflammatory, and neoplastic etiologies, accurate recognition of fibromatosis colli is important. Ultrasonography is generally preferred as the initial imaging modality because it can demonstrate characteristic SCM abnormalities without exposing the infant to ionizing radiation [2,3].

We present the case of a two-week-old female patient with a newly identified left-sided cervical mass ultimately diagnosed as fibromatosis colli. This case highlights the evolving clinical and sonographic appearance of fibromatosis colli, the importance of recognizing its relationship to the SCM, and the value of continued surveillance for subsequent cervical asymmetry.

## Case presentation

A two-week-old female patient presented to her pediatrician for a routine well-child examination. She had previously been evaluated in the pediatric office following discharge from the newborn nursery, at which time she was growing appropriately and had no concerning findings on physical examination.

The patient was born at 40 weeks and five days of gestation via spontaneous vaginal delivery to a 28-year-old G1P1 mother. She had a birth weight of 3.956 kg (8 lb 11.5 oz). Spontaneous rupture of membranes occurred approximately 33 hours and 37 minutes prior to delivery. Her Apgar scores were seven and nine at one and five minutes, respectively. She did not require resuscitation, and her subsequent newborn course was uncomplicated. She demonstrated normal newborn behavior with appropriate feeding, stooling, and urination. Her parents reported no concerns at the time of discharge.

At her initial outpatient newborn examination, no cervical mass or abnormal head positioning was noted. She continued to gain weight appropriately and was feeding without difficulty every two to three hours with an appropriate number of wet diapers. At her two-week well-child examination, she had no recent history of cough, congestion, respiratory distress, or feeding difficulty. Physical examination revealed an approximately 2 cm, firm, round, well-circumscribed mass on the left anterior neck that was non-tender to palpation and without overlying erythema. The mass was not easily mobile and appeared adherent to the underlying tissues; however, a definite association with or origin from the SCM could not be determined on physical examination. No additional masses, hepatomegaly, or splenomegaly were identified. Given the newly identified cervical mass in an otherwise well-appearing neonate, ultrasonography of the neck was obtained for further evaluation.

Ultrasonography demonstrated a 2.1 × 1.5 × 2.5 cm complex, circumscribed structure anterior to the left common carotid artery and internal jugular vein. The lesion demonstrated internal doppler vascularity and lacked a visible fatty hilum, raising consideration of a solid lesion or abnormal lymph node. No adjacent fluid collection or inflammatory changes were identified. The initial ultrasound interpretation did not identify a definite relationship between the sonographic abnormality and the SCM. Given the indeterminate sonographic appearance, further evaluation with contrast-enhanced computed tomography (CT) of the neck was recommended (Figure 1).

**Figure 1:**
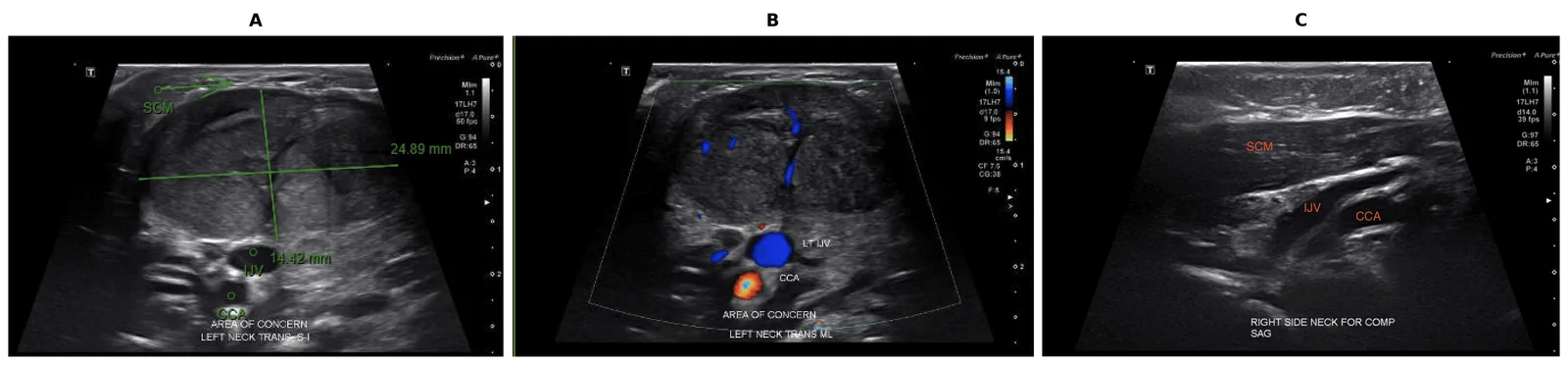
Ultrasonographic appearance of the left cervical mass (A) Transverse grayscale ultrasonographic image of the left neck demonstrating a complex, circumscribed mass anterior to the carotid vasculature. The mass measured 2.1 × 1.5 × 2.5 cm; (B) Color doppler ultrasonographic image of the left neck demonstrating internal vascularity within the area of concern; (C) Sagittal grayscale ultrasonographic image of the right neck obtained for comparison, demonstrating the normal contralateral sternocleidomastoid (SCM) muscle with typical echotexture. IJV: internal jugular vein; CCA: common carotid artery.

The patient was subsequently referred to a tertiary pediatric center for evaluation by pediatric otolaryngology. Contrast-enhanced CT of the neck demonstrated fusiform enlargement of the left SCM muscle, while the right SCM muscle was unremarkable. No discrete cervical mass, fluid collection, or abnormal enhancement was identified. There was no cervical lymphadenopathy by CT size criteria. The cervical vasculature and airway were unremarkable, and no abnormalities of the salivary or thyroid glands were identified. The imaging findings were considered consistent with fibromatosis colli of the left SCM muscle. The outside ultrasound was obtained for comparison during CT interpretation and was retrospectively interpreted as also demonstrating left fibromatosis colli.

Following diagnosis, the patient remained clinically stable with reassuring serial neck examinations. She remained afebrile and hemodynamically stable without respiratory compromise or difficulty with oral intake and continued to demonstrate appropriate weight gain. Given her clinical stability and the benign nature of the condition, no surgical intervention was indicated. She was discharged with close primary care follow-up and referrals for physical and occupational therapy due to the risk of torticollis.

Physical and occupational therapy evaluation at seven weeks of age demonstrated overall developmental skills within the expected range for her age. Torticollis was documented, with asymmetric cervical positioning characterized by rightward head rotation and leftward head tilt. Continued developmental physical or occupational therapy was recommended to address cervical muscle tightness, improve symmetry of head positioning and cervical range of motion, and support age-appropriate gross motor development. Caregiver education and a home program were incorporated into management to reinforce therapeutic strategies between visits.

At approximately 4.5 months following the initial diagnosis and three months after the initial therapy evaluation, parental report indicated improvement in cervical mobility and achievement of multiple therapeutic goals. The patient's mother reported equal head rotation to both sides, symmetric rolling, bilateral transfer of toys, and supported sitting. Progress regarding prone positioning and elevation of the head and chest on the forearms was not documented. The therapy assessment characterized the patient's torticollis as resolved based on parental report, and she was discharged from services after meeting the documented treatment goals. No follow-up imaging was performed to assess resolution of the SCM abnormality.

Parental consent was obtained for publication of this case.

## Discussion

Fibromatosis colli is a benign disorder of the SCM that most commonly becomes clinically apparent during the first several weeks of life. Wei et al. describe fibromatosis colli, or pseudotumor of infancy, as typically presenting with a firm, nontender neck mass before six weeks of age and note that previous literature supports development of the mass between two and four weeks after birth [2]. Lowry et al. similarly reported presentation between one and 3.5 weeks of age in a series of seven infants with fibromatosis colli [4]. This timing is consistent with the present case, in which no cervical abnormality was identified during the newborn period or initial outpatient examination, but a palpable mass was discovered at the two-week well-child visit. This delayed presentation is clinically important because a normal newborn examination does not exclude the subsequent development of fibromatosis colli.

The pathogenesis of fibromatosis colli remains incompletely understood. Proposed mechanisms include injury associated with difficult delivery or birth trauma, intrauterine positioning, impaired venous outflow within the SCM, and intrauterine or perinatal compartment syndrome [1,2]. However, the association with traumatic delivery is inconsistent. In the series by Wei et al., 60% of patients with available birth histories were delivered by uncomplicated spontaneous vaginal delivery, while 17.7% had documented birth complications [2]. These findings suggest that traumatic delivery alone cannot adequately explain the development of fibromatosis colli. In the present case, the patient was delivered vaginally following a prolonged interval between rupture of membranes and delivery but did not require resuscitation and had an otherwise uncomplicated neonatal course. Although this history is noteworthy, a causal relationship between the circumstances of delivery and the subsequent development of fibromatosis colli cannot be established.

Recognition of fibromatosis colli is important because the differential diagnosis of a neonatal cervical mass includes congenital, inflammatory, and neoplastic conditions [3]. Ultrasonography is the preferred initial imaging modality because it can demonstrate focal or diffuse enlargement of the SCM, is easily performed, and does not expose the infant to ionizing radiation [3]. Reported sonographic findings include fusiform enlargement or thickening of the affected SCM, synchronous movement of the lesion with the muscle on real-time imaging, and preservation of the fibrillar architecture of the muscle fibers [3,5]. Sonographic characteristics may also vary according to the stage of SCM involvement. Wang et al., in a study of congenital muscular torticollis, described local SCM thickening with weak or uneven echoes in early-stage disease and reported color Doppler blood-flow signals within or around the thickened SCM in some patients [6]. Although these findings were described in congenital muscular torticollis rather than specifically in fibromatosis colli, they demonstrate that doppler vascularity may occur in abnormal SCM tissue and should be interpreted in conjunction with the lesion's morphology and relationship to the muscle [6].

In the present case, initial ultrasonography demonstrated a 2.1 × 1.5 × 2.5 cm complex, circumscribed structure anterior to the left common carotid artery and internal jugular vein with internal doppler vascularity and no visible fatty hilum (Figure 1). These findings raised concern for a solid lesion or abnormal lymph node rather than establishing the diagnosis of fibromatosis colli. The initial ultrasound interpretation did not identify a definite relationship between the sonographic abnormality and the SCM, and the indeterminate appearance prompted further evaluation with contrast-enhanced CT. CT demonstrated fusiform enlargement of the left SCM without a discrete cervical mass, fluid collection, abnormal enhancement, or cervical lymphadenopathy, findings considered consistent with fibromatosis colli. The outside ultrasound was subsequently obtained for comparison during CT interpretation and was retrospectively interpreted as also demonstrating left fibromatosis colli.

The imaging course in this patient illustrates an important diagnostic consideration. Crawford et al. recommended ultrasonography as the initial imaging examination for fibromatosis colli and stated that CT should not be necessary when the clinical and sonographic findings are compatible with the diagnosis [3]. Wei et al. similarly emphasized the advantages of ultrasonography, including the absence of ionizing radiation, and reported that CT was performed in only 4.1% of their 170-patient cohort [2]. Cross-sectional imaging with CT or MRI may, however, be used when clinical findings are equivocal or atypical or when further characterization of the lesion is required [5]. In the present patient, concern for a vascular solid lesion or abnormal lymph node on the initial ultrasound prompted additional evaluation. Subsequent recognition that the sonographic abnormality represented the enlarged SCM emphasizes the importance of assessing the relationship of a neonatal neck mass to the SCM.

Fibromatosis colli and CMT are closely associated but are not synonymous. Fibromatosis colli refers to the fibrous SCM lesion, whereas CMT describes the associated clinical postural abnormality; the two have been proposed to represent a spectrum of pathologic changes involving the SCM [2]. Lowry et al. reported that five of seven infants with fibromatosis colli developed torticollis during their clinical course. With unilateral SCM contracture, the head may tilt toward the affected side with rotation toward the contralateral side [4]. The clinical evolution of the present patient followed this pattern. Although abnormal head positioning was not documented when the cervical mass was initially identified, torticollis was subsequently documented during physical and occupational therapy evaluation at seven weeks of age, with leftward head tilt and rightward head rotation. This progression illustrates the importance of continued surveillance even when torticollis is absent at the initial presentation of fibromatosis colli.

Management is predominantly conservative, and favorable outcomes are common. In the series by Wei et al., 136 of 159 patients with available follow-up experienced total resolution. Among the 22 patients with pseudotumor of infancy who had a mass at diagnosis and available follow-up, 21 experienced total resolution [2]. Lowry et al. reported complete resolution in five of seven patients and improvement in the remaining two; all seven were treated conservatively with physiotherapy [4]. Current evidence-based physical therapy guidelines recommend referral of infants with an SCM mass, postural preference, reduced cervical range of motion, or craniofacial asymmetry to a primary care provider and a physical therapist with expertise in infants as soon as the abnormality is identified [7]. Recommended first-choice physical therapy interventions include cervical passive range of motion when limited, active neck and trunk range of motion, symmetrical movement, environmental adaptations, and parent or caregiver education [7]. The 2024 clinical practice guideline also reports that earlier initiation of physical therapy is associated with better outcomes and shorter episodes of care [7].

The clinical course of the present patient was also favorable during conservative management. Following identification of torticollis, physical and occupational therapy focused on cervical mobility, symmetrical movement, and age-appropriate motor development. Approximately 4.5 months following diagnosis and three months after the initial therapy evaluation, parental report indicated equal bilateral head rotation and achievement of therapeutic goals involving symmetric rolling, bilateral hand use, and supported sitting. The therapy assessment characterized the torticollis as resolved based on parental report, although objective cervical range-of-motion measurements were not documented at the final assessment. No follow-up imaging was performed; therefore, clinical resolution of the torticollis should not be interpreted as documented resolution of the SCM abnormality.

This case demonstrates several clinically relevant features of fibromatosis colli. The cervical mass may become apparent only after an initially normal newborn examination, and its sonographic appearance may create diagnostic uncertainty. Recognition of the relationship between the sonographic abnormality and the SCM may facilitate diagnosis and help distinguish fibromatosis colli from other neonatal neck masses, potentially avoiding unnecessary additional imaging. Furthermore, CMT may become clinically apparent after identification of the initial SCM mass, emphasizing the importance of continued clinical surveillance and appropriate conservative management.

## Conclusions

Fibromatosis colli is a benign condition that should be considered in infants presenting with a cervical mass during the first weeks of life. Although ultrasonography is typically sufficient for diagnosis, variable sonographic findings may create diagnostic uncertainty. Careful attention to the relationship between the sonographic abnormality and the SCM may improve diagnostic confidence and potentially avoid additional imaging. CMT may become clinically apparent after identification of the initial SCM mass, making continued clinical follow-up important even when abnormal head positioning is not present at initial presentation. Conservative management, including physical therapy when indicated, remains the mainstay of treatment.
